# Effects of Pennyroyal (*Mentha pulegium* L.) Supplementation on production performance, egg quality traits, and biochemical parameters of blood and egg in laying hens at later stages of the production period

**DOI:** 10.1002/vms3.1031

**Published:** 2022-12-10

**Authors:** Amir Mosayyeb Zadeh, Seyyed Ali Mirghelenj, Peyman Hasanlou, Hossein Shakouri Alishah

**Affiliations:** ^1^ Department of Animal Science Faculty of Agriculture Urmia University Urmia Iran

**Keywords:** Antioxidant, egg cholesterol, hepatotoxic, laying hen, Pennyroyal, production performance

## Abstract

**Background:**

According to the promising outcomes acquired in recent studies that focused on using natural feed additives in animal nutrition, poultry nutritionists have also been interested in investigating these medicinal herbs' effects on poultry performance and egg characteristics. Pennyroyal (*Mentha pulegium* L.), a natural source of antioxidant, antimicrobial, anti‐inflammatory, etc. compounds, is recommended as a potential alternative for antibiotics, with similar benefits and no adverse effects on animal health or their products. Considerable effects have been reported on laying hens’ performance and their eggs’ internal and external traits by pennyroyal inclusion in their diets.

**Objectives:**

Due to the different results reported by pennyroyal inclusion in laying hens’ diets in recent limited studies, this study was designed to reinvestigate the impacts of pennyroyal (*Mentha pulegium* L.) supplementation (PS) on productive performance, egg quality traits, and biochemical compounds in blood and egg yolk in aged laying hens. The findings of this experiment may help for a better understanding of PS impacts on desired parameters, along with improving aged laying hens’ performance.

**Materials and Methods:**

A total of 144 Leghorn (Hyline‐W36 strain) laying hens (53 weeks old) were used to investigate the effects of PS on performance, egg quality, and biochemical parameters of blood and egg yolk in old laying hens. The animals were distributed into three treatment groups; including a control diet (without PS), 0.5%, and 1% PS diets with six replicates and eight birds per replicate.

**Results:**

The results indicated that the egg production rate (except for week eight) and egg mass were significantly reduced by PS in the laying hens’ diet during the first four weeks and the total period of the experiment (P<0.05). However, the feed conversion ratio increased by PS (P<0.05). Based on the egg quality trait evaluations (week 4), the yolk percentage, yolk height, and yolk index reduced, while shell‐breaking strength represented a significant increase in PS groups (P<0.05). However, shell thickness and albumen percentage reduced and increased by 1% PS, respectively (P<0.05). At week eight, the Haugh unit reduced while the albumen pH increased by PS (P<0.05). The findings revealed a decline in the egg density in 1% PS groups (P<0.05). In the case of serum biochemical parameters, alanine aminotransferase increased, whereas malondialdehyde (MDA) levels reduced in the PS groups (P<0.05). Moreover, the triglyceride levels of both serum and egg yolk (accompanied by the very low‐density lipoprotein level of serum) decreased by PS (P<0.05).

**Conclusions:**

In conclusion, PS reduced serum and egg yolk triglyceride levels, accompanied by serum MDA, along with reducing production performance.

## Graphical abstract

The graphical abstract image represents a pennyroyal leave and its grounded form which was supplemented in laying hens’ diet in order to evaluate its impact on the production performance, egg quality, and biochemical contents of blood and eggs in the current study.

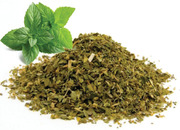

## 1 INTRODUCTION

Laying hens, especially the older ones, are highly prone to oxidative stresses that strongly affect their production performance (Kirk et al., 1980). Therefore, using antioxidants or reactive oxygen species scavengers, including N‐acetylcysteine or catalase (Denamur et al., 2011), to prevent the adverse effects of oxidative stress is beneficial. On the other hand, most countries have banned the use of antibiotics above the therapeutic dosage due to concerns about antibiotic residence in animal products and thus the development of antibiotic resistance in humans. Hence, finding an appropriate antibiotic alternative with similar benefits and no adverse effects has gained considerable interest (Khan et al., 2012). Researchers have reported the beneficial impacts of medicinal herbs’ essential oils (EOs) and their powder in animal production while looking for a natural antibiotic alternative (Windisch et al., 2008). Accordingly, some individual features of these phytogenic plants include appetite stimulation, feed conversion ratio (FCR) improvement, and body weight gain increase (Bahadori et al., 2013; Christaki et al., 2011; Saleh et al., 2014). Furthermore, these plants increase endogenous enzyme secretion and immune responses (Grashorn, 2010; Windisch et al., 2008), modulate the microbial population of the gut (Vispute et al., 2019; Windisch et al., 2008), and improve nutrient digestion and absorption (Hajiaghapour & Rezaeipour, 2018; Saleh et al., 2014). In addition to the antioxidant effect of medicinal herbs on laying hens’ production performance, some recent studies have proved that phytoestrogenic compounds in these plants are responsible for performance enhancement in aged laying hens (e.g., Saleh et al., 2019; Shi et al., 2013).

Pennyroyal has been considered a potential candidate as a natural alternative to antibiotics and synthetic antioxidants due to its antimicrobial and antioxidant properties (Ahmed et al., 2018). Pennyroyal, a common name for *Mentha pulegium* L., belongs to the Labiatae family and is a native species of Europe, North Africa, and Asia (Chalchat et al., 2000). A wide range of polyphenolic compounds with different biological functions has been recognized in the phytogenic plants of this family (Erhan et al., 2012). This medicinal herb has been used in traditional medicine due to its anti‐inflammatory, anti‐flatulence, pain‐killer, and antispasmodic properties (Kamkar et al., 2010) in healing common colds, sinusitis, cholera, bronchitis, and tuberculosis disorders (Ahmed et al., 2018); however, in some regions, it has been applied as a pest eradication poison due to its toxic compounds (Arjomandi et al., 2011). A vast range of variations has been observed concerning the chemical composition of pennyroyal EOs. Pennyroyal has been reported to contain 1–2% EO, and pulegone is its dominant (60–90%) component (Barnes et al., 2002), whereas in another study, 1,8‐cineole has been determined as the most dominant component of its EOs (Mohammadi, 2020). One study demonstrated the toxic effects of pulegone on the liver (Arjomandi et al., 2011), and it has been recognized that its molecular formula is C10H16O (Sullivan et al., 1979).

A small number of studies have been conducted to investigate the effects of pennyroyal supplementation (PS) on laying hens, and the obtained results are highly confusing. For instance, a recent study reported that the PS did not affect feed intake (FI) and most of the egg quality traits. However, it significantly improved FCR, egg weight (EW), egg production (EP) percentage, and shell breaking strength (SHS) (Aydın & Bölükbaşı, 2020). In a similar study, adding 0.5% PS to young laying hens’ diets increased FCR while decreasing EP and EM. Additionally, the shell thickness (SHT) was adversely affected by PS (Nobakht et al., 2011). In another investigation, using pennyroyal powder and extract supplementation in laying hens’ diet, it was reported that pennyroyal powder inclusion of up to 1.5% could improve production performance, the immune system, egg quality traits, and blood biochemical parameters. However, using pennyroyal extracts up to 0.2–0.3% reduced production performance and increased both the egg density (ED) and Haugh unit (HU) (Paymard et al., 2013). Besides, positive impacts were reported regarding blood biochemical parameters by applying pennyroyal extracts (Paymard et al., 2013). Arjomandi et al. (2011) found that 2% of PS, individually or combined with probiotics, might reduce production performance.

As mentioned earlier, limited studies have examined the effects of PS on laying hens, especially older hens. Therefore, the present experiment aimed at investigating the impacts of different levels of pennyroyal powder supplementation (slightly lower levels compared to previous studies) on performance, egg quality traits, and biochemical parameters of serum and egg yolk in old laying hens to find an appropriate PS level in laying hens’ diet.

## 2 MATERIAL AND METHODS

### 2.1 House preparation and treatments

In general, 144 laying hens (Hy‐line‐W36) at 53 weeks of age were randomly assigned to three treatment groups to investigate the effects of PS (*Mentha pulegium* L.) on productive performance, egg quality traits, and biochemical parameters of serum and egg yolk in the present experiment. The treatment included six replicates and eight birds per replicate (with similar body weights ±5% of average body weight). Hens were purchased from the nearest commercial layer farm and housed in commercial layer cages (80 × 80 × 115 cm) with free access to freshwater using a nipple drinking system and fed a 100 g diet in two daily meals (7:00 a.m. and 12:00 p.m.) during the adaptation and experimental periods (50 g for each meal). Experimental diets included a control diet without PS and two diets with PS in amounts of 0.5 and 1% (Table 1). The diets were corn‐soybean meal‐based and formulated according to the nutritional recommendations of the Hy‐line W36 strain guidelines, and they were fed for 8 weeks after the adaptation period. During the adaptation period, which lasted for 2 weeks, the birds were fed the control diet with free access to water. A lightning program of 14L: 10D was set with an intensity of 30 lx for the light period and a maximum of 5 lx for the dark period. The house's ambient temperature and humidity were maintained at 20–25°C and 40–50%, respectively.

**TABLE 1:** Feed ingredients and composition of experimental diets

**Feed ingredients (% as fed)**	**Control diet**	**0.5% Pennyroyal**	**1% Pennyroyal**
Corn	55.88	58.36	57.63
Soybean meal (44%)	24.64	24.75	24.70
Wheat bran	2.5	3	4
Soy oil	2	1.27	1.46
Dicalcium phosphate (DCP)	1.51	1.51	1.51
Oyster shell	12.23	12.06	12.06
Common salt	0.25	0.26	0.26
Sodium bicarbonate	0.15	0.15	0.15
DL‐Methionine	0.19	0.19	0.19
L‐Lysine HCl	0.04	0.05	0.05
Vitamin premix	0.3	0.3	0.3
Mineral premix	0.3	0.3	0.3
Pennyroyal powder	–	0.5	1

**Nutrient composition**			
ME (kcal/kg feed)	2.65	2.65	2.65
Crude protein (%)	16.2	16.2	16.2
Calcium (%)	5	5	5
Available phosphor (%)	0.41	0.41	0.41
Crude fiber (%)	3.3	3.9	4.5
Sodium (%)	0.18	0.18	0.18
Methionine (%)	0.45	0.45	0.45
Methionine + Cysteine (%)	0.73	0.73	0.73
Lysine (%)	0.85	0.85	0.85
Threonine (%)	0.55	0.55	0.55
Tryptophan (%)	0.22	0.22	0.22
DEB (Meq/kg)	205	201	199

Supplied vitamins per kilogram of diet: Vit A: 10000 IU; Vit D3: 2500 IU; Vit E: 10 IU; Vit B1: 2.2 mg; Vit B2: 4 mg; Vit B3: 8 mg; Vit B6: 2 mg; Vit B9: 0.56 mg; Vit B12: 0.015 mg; Choline 200 mg.

Supplied minerals per kilogram of diet: Mn: 80 mg; Fe: 50 mg; Zn: 60 mg; Cu: 12 mg; Sodium Selenite: 0.3 mg.

Abbreviations: DEB, Dietary electrolyte balance.

### 2.2 Pennyroyal powder

The aerial parts of the pennyroyal (*Mentha pulegium* L.) were purchased fresh from a local grocery store, air‐dried, and finally ground for use in the laying hens’ diet and measuring the amount of its dominant polyphenolic components, especially pulegone. The chemical composition and EOs of the applied pennyroyal powder in the present experiment were measured by a chemical method described by Sparkman (Sparkman, 1997) using gas chromatography‐mass spectrometry (GC/MS). The results indicated that the pennyroyal powder contained 17.11, 14.54, 14.76, 4.43, 3.80, 7.16, 5.63, and 4.91% pulegone, 1,8‐cineole, piperitone, cis‐piperitone oxide, piperitone oxide, menthol, isomentone, and p‐mentone, respectively. Furthermore, four samples of dry pennyroyal powder were analyzed for dry matter (DM), gross energy (GE), ether extract (EE), crude protein (CP), and crude fiber (CF) according to (AOAC, 1990). The respective values for the determined nutrient composition were 88.6% (DM), 1500 kcal/g (GE), 4% (EE), 11% (CP), 46.60% (CF), 0.85% (calcium), and 0.2% (phosphorous).

### 2.3 Production performance

The eggs were collected two times a day, and the egg number and the mean EW were recorded at the end of each day. The EM, FCR, and FI were calculated weekly. Furthermore, the EP percentage was evaluated by dividing the total produced eggs by each replicate by its hen day. The EM was also estimated by multiplying the mean EW of each replicate by its EP percentage. Additionally, weekly FI was divided into the EM to estimate the FCR.

### 2.4 Egg quality measurement

The internal and external quality traits of the egg were investigated at weeks four and eight of the experiment. For this purpose, two eggs were collected from each replicate and stored at room temperature for 24 h to chill. The weight of the EW and egg components, including egg yolk, egg white, and eggshell, was measured using a digital scale (0.01 g; KEB 602, China) to estimate the percentages of the yolk (YP), albumen (AP), and eggshell (SHP) accompanied with the HU. The yolk diameter (YD) was measured by a digital caliper (0.01 mm; BakingWin, China) to evaluate the yolk index (YI). The ED was measured by providing an aqueous solution (distilled water) added with different levels of salt (NaCl) according to the method of Florida University (Butcher & Miles, 1991). A force reader machine (Ogawa Seiki 020603, Co., Ltd., Tokyo, Japan) was provided by the Agricultural Jihad Office of West Azerbaijan Province to determine the SHS of the eggs. The egg white and egg yolk heights were measured using a Haugh meter for the HU and YI evaluations, respectively. The egg white height values were recorded wherever the tip of the altimeter of the Haugh meter hit the egg white (1 cm around the yolk). The following equation was used to calculate the Haugh unit (Haugh, 1937):

$$\mathrm{HU}=\log\left(\mathrm{AH}-1.7EW^{0.37}+7.57\right)$$

The Rosh fan color scale was applied to evaluate the egg yolk color. The YI was calculated by dividing the yolk height (YH) into the YD (Funk, 1948). The egg yolk and egg white were separated, and then their pH was measured by a pH meter (Microcontroller MTT 65, Japan) after mixing the egg white or yolk with distilled water in a ratio of 1:9 and subsiding the produced foam (Funk, 1948). An outside micrometer (0.01 mm; Model Outside meter YP001, Japan) was employed to measure the eggshell thickness from the top, middle, and bellow (or the air‐cell area) sections. Finally, the average of these sections was computed and considered as the eggshell thickness. It should be noted that before these measurements, the eggshells were weighed after washing and drying at room temperature for 12 h, and then in a 65°C oven for 72 h.

### 2.5 Blood and egg yolk biochemicals

The serum samples were obtained by taking blood samples from the wing veins of the two birds in each replicate using 5 ml plastic syringes. The blood samples were kept at room temperature for 8 h, and the obtained serum was cautiously poured into 2 ml microtubes to be stored at ‐20°C until performing laboratory experiments. A 100 mg of egg yolk was weighed by a digital scale (0.001 g; model JT3003D, China), mixed with 2.5 ml of NaOH solution 0.05N, and neutralized with 2.5 ml of HCl 0.25 N solution (after 24 h). Serum and egg yolk lipids were analyzed at a 550 nm wavelength by an autoanalyzer machine (model Technicon RA1000) using the Pars Azmoun kits according to the enzymatic method described in their instruments. The other serum biochemical parameters, including the levels of liver enzymes such as alanine aminotransferase (ALT), alkaline phosphatase (ALP), and aspartate aminotransferase (AST), as well as total protein, uric acid, malondialdehyde (MDA), and total antioxidant capacity (TAC), were measured by the Pars Azmoun kits briefly described in a previous study (Mosayyeb Zadeh et al., 2021). As a short description, the serum TAC level was determined by Randox Total Antioxidant Control Cat. No. NX2331, kits, and their instruments at 600 nm of wavelength produced by a temperature‐controlled spectrophotometer. A solution containing 500 µl of a serum sample, 3 ml of phosphoric acid (1%), and 1 ml of thiobarbituric acid (0.67%) was mixed and boiled for 45 min in a bain‐marie to evaluate the malondialdehyde (MDA) content. In addition, 3 ml of normal butanol was added to the obtained solution in the test tube and mixed (for a maximum of 2 min) to prepare for centrifugation for 10 min at 3000 rpm. The obtained supernatants were exposed to the 532 nm wavelength of light absorbance, and finally, the results were compared with the standard curve (normal butanol). Liver enzyme (ALT, AST, and ALP) levels in the birds’ serum were determined using the materials and methods provided by Pars Azmoon Company, Tehran, Iran. In this case, ALT and AST levels were estimated at the wavelengths of 600 and 340 nm after preparing the desired solutions according to the procedures provided by the above‐mentioned company. Similarly, the ALP level was measured by the method of (Thomas, 1998) and Pars Azmoon kits.

### 2.6 Statical analysis

All the obtained data were statistically analyzed by the general linear model using SAS software, version 9.2 (SAS, 2009). The Tukey's test was used to determine significant differences among the means, and P‐values less than 0.05 were considered statistically significant. The applied statistical model in this experiment is as follows:

$$Y_{\mathrm{ij}}=\mu +T_{i}+\varepsilon_{\mathrm{ij}}$$

where Y_ij_, µ, T_i_, and ε_ij_ represent observation, mean of observations, treatment effect, and experimental error of each observation, respectively.

## 3 RESULTS

### 3.1 Production performance

The results of the effects of PS in the laying hens’ diet on their productive performance are presented in Table 2. Based on the findings, the EP percentage was significantly reduced by PS in the laying hens’ diet during the first four weeks of the experiment (P<0.05); however, it was not affected during the second four weeks. The evaluations indicated that the EP percentage was significantly decreased during the total period of the experiment compared to the control group (P<0.05). Likewise, the EM represented a significant reduction in laying hens fed with the diet supplemented with pennyroyal powder during the first and second four weeks of the experiment, as well as over the entire period (P<0.05). Conversely, the FCR was significantly increased by PS in the laying hens’ diet in comparison to the control group (P<0.05). The mean EW and FI were not influenced by the inclusion of pennyroyal in the laying hens’ diet. Furthermore, the mean body weight gain and viability were not significantly different among the experimental groups. The mean body weight gains (30 ± 3.9 g) and viability (98% ± 0.9) of the hens throughout the experiment were also evaluated at the end; however, since the means were not significant, the related data are not reported in the tables.

**TABLE 2:** Effects of different levels of pennyroyal supplementation on production performance of laying hens

	**Egg production (%)**			**Egg weight (g)**			**Egg mass (g)**			**Feed intake (g)**			**FCR (g: g)**		
**Pennyroyal (%)**	Week 0‐4	Week 4–8	Total period	Week 0‐4	Week 4–8	Total period	Week 0‐4	Week 4–8	Total period	Week 0‐4	Week 4‐8	Total period	Week 0–4	Week 4–8	Total period
Control	66.96^a^	67.41	67.19^a^	63.66	64.73	64.20	42.62^a^	43.63^a^	43.13^a^	99.75	99.50	99.62	2.34^b^	2.28^b^	2.31^b^
0.5	62.27^b^	64.28	63.28^b^	62.28	62.71	62.49	38.79^b^	40.30^b^	39.54^b^	99.50	99.50	99.50	2.56^a^	2.47^a^	2.51^a^
1	60.71^b^	64.29	62.50^b^	62.90	62.95	62.93	38.17^b^	40.46^b^	39.32^b^	99.00	99.50	99.25	2.59^a^	2.46^a^	2.52^a^
SEM	0.761	0.929	0.712	0.656	0.691	0.551	0.389	0.666	0.365	0.520	0.500	0.469	0.027	0.044	0.028
P‐value	<0.01	0.064	<0.01	0.371	0.131	0.129	<0.01	0.011	<0.01	0.601	0.990	0.850	<0.01	0.027	<0.01

*Note*: ^a, b^Means within same column with different letters differ significantly (P<0.05).

Abbreviations: FCR, feed conversion ratio (g feed consumed: g produced egg).

### 3.2 Egg quality traits

Table 3 summarizes the results of the effects of pennyroyal inclusion in the laying hens’ diet on the internal and external quality traits of the eggs. According to the obtained data, the YP, YH, and YI demonstrated a significant decline in both levels of PS, in the laying hens’ diet during the first four weeks of the experiment compared to the control group (P<0.05). The eggshell thickness was only significantly reduced in the group of birds fed with diets containing 1% PS during this period (P<0.05). Contrarily, AP was significantly increased by 1% pennyroyal inclusion in the diet (P<0.05). Furthermore, SHS showed a significant increase in the groups of the birds fed with diets including different levels of PS during this period in comparison with the control group (P<0.05). During the second four weeks of the experiment, the HU reduced significantly, while the ApH was increased by PS in the laying hens’ diet (P<0.05). Eventually, the ED represented a reduction in the group of birds fed with diets containing 1% pennyroyal powder compared to the control group (P<0.05).

**TABLE 3:** Effects of different levels of pennyroyal supplementation on quality traits of egg

**Pennyroyal (%)**	**YP (%)**	**AP (%)**	**SHP (%)**	**SHT (mm)**	**HU**	**YH (mm)**	**ApH**	**YpH**	**ED**	**SHS (kg/m^2^)**	**YC**	**YI**
Week 4												
Control	28.84^a^	65.53	8.62	0.423^b^	84.67	17.06^a^	8.12	6.88	1.0875	1.60^b^	8.75	39.37^a^
0.5	25.69^b^	66.10	9.19	0.445^ab^	87.93	15.56^b^	8.25	6.85	1.0837	3.45^a^	8.25	35.65^b^
1	23.82^b^	67.69	8.48	0.478^a^	84.96	15.47^b^	8.16	6.90	1.0837	2.98^a^	8.87	35.67^b^
SEM	0.830	0.825	0.273	0.013	1.965	0.370	0.108	0.131	0.002	0.313	0.216	0.883
P‐value	<0.01	0.367	0.170	0.028	0.444	<0.01	0.667	0.952	0.355	<0.01	0.121	<0.01
Week 8												
Control	27.96	63.28	8.75	0.437	83.85^a^	15.85	7.58^b^	6.73	1.0937^a^	2.43	8.75	35.97
0.5	29.95	61.28	8.76	0.416	73.76^b^	17.41	8.09^a^	7.01	1.0862^ab^	2.33	8.50	40.32
1	29.14	62.17	8.68	0.427	73.37^b^	15.47	8.39^a^	7.09	1.0850^b^	2.18	8.87	35.60
SEM	0.820	0.827	0.240	0.011	2.662	0.653	0.109	0.108	0.002	0.350	0.312	1.510
P‐value	0.250	0.254	0.969	0.456	0.045	0.108	<0.01	0.070	0.018	0.879	0.692	0.070

*Note*: ^a, b^Means within same column with different letters differ significantly (P<0.05).

Abbreviations: YP, Yolk percentage; AP, Albumen percentage; SHP, Shell percentage; SHT, Shell thickness; HU, Haugh unit; YH, Yolk height; ApH, Albumin pH; YpH, Yolk pH; ED, Egg density; SHS, Shell breaking Strength; YC, Yolk color index; YI, yolk index; SEM, standard error of mean.

### 3.3 Blood and egg yolk biochemical components

Tables 5 and 4 provide the obtained data by evaluating the biochemical parameters of the blood and egg yolk of laying hens fed diets supplemented with different levels of pennyroyal. The levels of ALT in the serum of the birds fed PS‐containing diets increased significantly (P<0.05), as shown. However, the serum MDA level was significantly reduced by supplementing the laying hens’ diet with different levels of pennyroyal powder compared to the control group (P<0.05). The evaluation of the level of lipids indicated that the triglyceride levels of both serum and egg yolk (accompanied by very low‐density lipoprotein [VLDL] in the serum) demonstrated a significant decline by PS (P<0.05); however, their cholesterol levels remained unchanged.

**TABLE 4:** Effects of different levels of pennyroyal supplementation on serum biochemical

**Pennyroyal (%)**	**Uric acid (mg/dl)**	**Total protein (g/ml)**	**Albumin (g/ml)**	**ALT (IU/L)**	**AST (IU/L)**	**ALP (IU/L)**	**TAC (mmol/l)**	**MDA (µg/ml)**
Control	4.07	5.65	2.62	36.00^b^	243.2	782.5	1.29	3.42^a^
0.5	4.31	5.67	2.32	54.05^a^	237.5	857.25	1.58	1.86^b^
1	4.08	6.00	2.40	61.25^a^	235.0	915.25	1.56	1.84^b^
SEM	0.426	0.470	0.166	6.33	12.06	45.17	0.120	0.226
P‐value	0.901	0.844	0.446	0.044	0.885	0.170	0.279	0.001

*Note*: ^a, b^Means within same column with different letters differ significantly (P<0.05).

Abbreviations: ALT, Alanine transaminase; AST, Aspartate transaminase; ALP, Alkaline phosphatase; TAC, Total antioxidant capacity; MDA, Malondialdehyde; SEM, standard error of mean.

## 4 DISCUSSION

This study investigated the effects of different levels of pennyroyal powder supplementation in laying hens on performance, egg quality traits, and biochemical parameters of blood and egg yolk at slightly lower levels compared to previous studies to determine a nontoxic level of pennyroyal powder for poultry nutrition, especially laying hens. For this purpose, the pennyroyal herb was purchased fresh, and its most common bioactive compounds were measured using GC/MS (Sparkman, 1997). The result revealed that the bioactive compounds of the applied pennyroyal powder were relatively similar to those of the pennyroyal powder that was used by Mohammadi (2020). However, due to limited examinations, there is conflicting information about the effects of PS in laying hens. The results obtained in this study demonstrated that PS in the laying hens’ diet significantly reduced the EP percentage during the first four weeks of the experiment, along with the total period of the study. The EM is an index that is based on the EP percentage and EW; therefore, a significant reduction in the EP percentage while the EW remained unchanged, resulted in a remarkable alleviation of the EM (the formula for calculation described previously). It is well known that the FCR is an index that depends on the EM and FI; accordingly, it is believed that the EM reduction (while the FI remained unchanged) throughout the study may have resulted in an elevation of the FCR. Therefore, any factor that may reduce the EP percentage will decrease these parameters as well. Thus, the reason for these changes might be attributed to any potential factor that could affect the EP percentage.

According to previous studies, the pennyroyal powder usually contains 1–2% EOs, and its most dominant component is pulegone, which might include 60–90% of the EOs (Barnes et al., 2002). Pulegone, with the chemical formula C10H16O, has also been found in the EOs of other medicinal herbs such as Nepeta cataria (catnip) and Mentha piperita (Sullivan et al., 1979). In line with our findings, some previous studies indicated that PS might reduce the production performance of laying hens due to the hepatic damage caused by the pulegone content of pennyroyal (e.g., Arjomandi et al., 2011; Nobakht et al., 2011; Paymard et al., 2013). It was reported that pulegone is a hepatotoxic component that exists in pennyroyal EOs and could potentially metabolize and convert to toxic compounds such as menthofuran (EC, 2002). Moreover, Arjomandi et al. (2011) employed dried pennyroyal to eradicate the pests in colonial Virginia due to its toxic feature. However, the findings of some studies contradict those of the present study, indicating that PS could significantly improve the production performance of both laying hens and broiler chickens (e.g., Aydın & Bölükbaşı, 2020; Erhan et al., 2012; Nobakht et al., 2011).

In this study, the biochemical compounds of blood were evaluated to find out if the pulegone content of the applied pennyroyal powder could impair hepatic cells. The result showed that ALT was significantly increased in the serum of the birds fed diets supplemented with different levels of pennyroyal powder, implying that hepatic cell injuries might be responsible for the reduction in the production performance of the laying hens in the present experiment. Liver injuries are widely studied by measuring hepatic enzyme levels in the blood. In this respect, ALT is thought to be the most common marker that is used as a regulatory and clinical tool for hepatic cell damage detection (Senior, 2012). As previously mentioned, the pulegone content of the pennyroyal powder used in the present experiment was high (17.11%). Therefore, it would be expected that supplementing the laying hens’ diet with the pennyroyal powder, which contains high pulegone content for a long time (8 weeks), might damage the hepatic cells and result in both reduced production performance and increased ALT in the serum. In contrast with these findings, some previous studies reported no significant changes in serum ALT levels by PS in the laying hens’ diet (e.g., Aydın & Bölükbaşı, 2020; Bolukbasi et al., 2018); this is supposed to be due to the higher concentration of pulegone and the other hepatotoxic compounds of the applied pennyroyal powder in the current work. Consequently, more investigations are needed to ensure the hepatotoxic effects of pennyroyal powder, especially histological studies that may determine the morphological changes of liver cells after PS in poultry diets.

Medicinal plants, including pennyroyal (*Mentha pulegium* L.), have gained extensive attention due to their antioxidant properties caused by their polyphenolic compounds. However, it has been reported that polyphenolic compounds might disrupt lipid digestion and absorption in the small intestine and subsequently reduce blood lipid levels, which might be responsible for decreasing EP performance (Ikeda et al., 1992). It was also stated that the yolk and EP mainly depend on the lipids, which are derived from the feed and transported by lipoprotein from the liver to the ovary (Lin et al., 2019; Schneider, 2009). Therefore, it would be expected that a reduction in blood lipids might be responsible for decreasing EP, EW, EM, and finally increasing FCR. Hence, reduced lipid levels in the blood beyond the free radical scavenging property of the polyphenolic compounds of pennyroyal might have potentially reduced the lipid oxidation, and thus, it might be one of the probable reasons for reduced MDA (a final metabolite of the lipid oxidation process) in laying hens’ blood. This assumption is well supported by previous studies that have attributed both the improvement in antioxidant status and the reduction in serum MDA to the hypocholesterolemic effect of phytoestrogens in herbal plants (Saleh et al., 2019). In agreement with these findings, previous investigations suggested that the antioxidant effects of pulegone and menthone might be responsible for the reduction of serum MDA by using pennyroyal powder in the birds’ diet (e.g., Çöteli et al., 2013; Kamkar et al., 2010; Ruberto & Baratta, 2000). Furthermore, Aydin & Bolukbasi (2020) concluded that PS in the laying hens’ diet increased their superoxide dismutase activity while reducing the MDA level in the serum. They further indicated that phenolic compounds reduced the formation of reactive oxygen species by binding free radicals to metal ions and thus decreased lipid oxidation.

As mentioned earlier, the results demonstrated that the yolk‐related characteristics of the eggs were mainly affected by PS during the first four weeks of the experiment. As mentioned above, reduced serum lipids might be a probable reason for decreasing the YP, YH, and therefore YI. These findings are in contrast with those of previous ones, reporting no significant impacts on these parameters by PS (Aydın & Bölükbaşı, 2020; Nobakht et al., 2011; Paymard et al., 2013). However, in the case of SHT and SHS, the results conform to those of previous studies (Aydın & Bölükbaşı, 2020; Nobakht et al., 2011). Nobakht et al. (2011) found that calcium supply per egg remains constant, while the EP represents a reduction; accordingly, it may increase calcium deposition per egg and SHT, thus probably increasing the SHS power. On the other hand, in line with the present findings, a serum calcium enhancement due to medical plants such as cumin seed supplementation in the laying hens’ diet could improve egg‐shell traits by shell classification augmentation (Saleh et al., 2020). In this regard, former scientists (e.g., Gu et al., 2013; Saleh et al., 2019) attributed the serum calcium increments to the effects of phytogenes found in herbal plants (flaxseed and fenugreek seed). Moreover, the regulatory impact of phytogenies on estrogen receptors (both α and β) in the shell glands of the uterus, which in turn influences the activity of carbonic anhydrase, has been proposed as another probable reason for shell trait improvements (Wistedt et al., 2012). Interestingly, during the second four weeks, only the HU, ApH, and ED parameters were affected by using pennyroyal powder supplementation in the laying hens’ diet. Previously, it was indicated that the phenolic compounds of medicinal plants might reduce protein production and secretion, especially in the hens’ oviduct (Goñí et al., 2007). Given that the HU parameter is dependent on the egg white thick protein, a reduction in egg white protein production and secretion might potentially reduce the HU and impair egg quality traits. Furthermore, another study reported a strong relationship between ApH and HU (e.g., Ahn et al., 1999). It was stated that increased ApH might have reduced protein bindings, especially those among the ovomucin and lysozyme of albumen, and thus reduced the HU. Additionally, they suggested that increased ion exchange between the egg yolk and egg white due to the oxidation of the yolk perivitelline membrane lipids might be responsible for both the pH alteration and thus a reduction in the HU. Considering that the ED is directly related to the HU, it is assumed that the low ED in the groups of the birds fed with pennyroyal‐supplemented treatments might be due to the low HU of their eggs. This finding is in conformity with that of Paymard et al. (2013), representing that the reduced calcium deposition in eggshells due to reduced FI might be responsible for reduced ED; nonetheless, the FI remained unchanged in the present experiment.

In the case of serum and egg yolk lipids, the effects of pennyroyal powder inclusion in the laying hens’ diet in the current study revealed that the cholesterol levels of both serum and egg yolk were significantly reduced due to the PS. In addition, the results showed a reduction in the VLDL level of the serum. These findings are in complete agreement with those of previous studies (e.g., Mohammadi, 2020; Paymard et al., 2013). As discussed above, it was indicated that the inclusion of phenolic compounds in herbal plants may potentially disturb lipids, especially biliary acids, absorption, and reabsorption processes (Ikeda et al., 1992). These comments have been proven in a recent study, in which the researchers showed that the phenolic compounds of the medicinal plant, especially the EOs of dill, may disrupt the biliary acids’ intestine‐liver cycle by binding them and preventing their absorption from the gut (Torki et al., 2018). Furthermore, in an earlier study, the researchers highlighted that the lipid concentration in blood may reduce due to the inhibitory effects of the EOs of herbal plants on the activity of the key enzymes in lipid metabolisms, including cholesterol‐7 hydroxylase fatty acid synthase and 3‐Hydroxy‐3‐Methylglutaryl‐CoA (HMG‐CoA) reductase (Qureshi et al., 1983; Saleh et al., 2015). Using cumin seed, Saleh et al. (2020) reported similar results, and the changes were thought to be because of the active compounds in the cumin seed, which potentially repress the function of cholestrogenic and lipogenic enzymes in hepatic cells, including glucose‐6‐phosphatase dehydrogenase, malic enzyme, and fatty acid synthesis (Chi et al., 1982). Additionally, it has been well documented that there is a direct relationship between triglyceride and VLDL levels in the blood. The triglyceride level in the blood is approximately five times higher than that of VLDL (Friedewald et al., 1972). Both VLDL and vitellogenin are the main precursors of blood lipoproteins, and their hepatic synthesis is highly affected by estradiol‐17β (Sturkie, 2000). Saleh et al. (2019), in line with previous studies (Dusza et al., 2006), confirmed a direct relationship between dietary phytogenic compounds and blood estradiol‐17β levels in old laying hens and concluded that the improved EP performance in the birds fed flaxseed and fenugreek seed may be caused by increased estradiol‐17β levels in the blood due to the estrogen receptor‐β mRNA upregulation in liver cells. Unfortunately, the blood estradiol‐17β level was not measured in the present experiment. However, according to the reductions in both serum VLDL and yolk triglyceride (Table 5), it is assumed that the inhibitory effects of pennyroyal EOs, especially pulegone, on lipid absorption and the activity of the lipid metabolism enzymes are probably responsible for low lipid levels in the blood and egg yolk, which may affect production performance.

**TABLE 5:** Effects of different levels of pennyroyal supplementation on serum and egg yolk cholesterol and triglyceride

	**Yolk**		**Serum**		
Pennyroyal (%)	Triglyceride (mg/g)	Cholesterol (mg/g)	Triglyceride (mg/dl)	Cholesterol (mg/dl)	VLDL (mg/dl)
Control	317.7^a^	28.25	2680.3^a^	572.50	536.0^a^
0.5	281.2^b^	28.00	2182.0^b^	493.75	435.5^b^
1	292.2^b^	29.50	2042.9^b^	481.50	408.5^b^
SEM	8.58	1.19	117.74	38.12	23.51
P‐value	0.042	0.314	<0.01	0.243	<0.01

*Note*: ^a, b^Means within same column with different letters differ significantly (P<0.05).

Abbreviations: VLDL, very low density lipoprotein; SEM, standard error of mean.

## 5 CONCLUSION

In general, pennyroyal (*Mentha pulegium* L.) powder supplementation in the laying hens’ diet reduced production performance and egg quality traits probably due to its phenolic compounds/EOs, especially pulegone, which has a hepatotoxic effect on liver cells. This finding was well proved by the serum ALT level increment in laying hens fed PS diets in the present investigation. Furthermore, the reduced serum and egg yolk lipids accompanied by the elevated MDA level were probably due to the same reasons. Thus, according to the probable hepatic injuries caused by the phenolic compounds of the pennyroyal in the present study, it is not recommended to use this herbal plant, especially from the same source, which contains more hepatotoxic compounds compared to previous examinations, in the laying hens’ diet, especially the old ones. Further investigations are required to ensure the exact mechanism of the hepatotoxic effects of pennyroyal and the histological changes that may be caused by its polyphenolic compounds.

## AUTHOR CONTRIBUTIONS

**Seyyed Ali Mirghelenj**: Conceptualization; funding acquisition; and supervision. **Amir Mosayyeb Zadeh**: Data curation, formal analysis; investigation; project administration; writing – original draft; and writing – review & editing. **Hossein Shakouri Alishah**: Resources; funding acquisition. **Peyman Hasanlou**: Resources, funding acquisition.

## CONFLICT OF INTEREST

The authors of this manuscript confirm that they have no conflicting interests to disclose, affecting their research work. Also, the accuracy and completeness of the submitted manuscript are under the corresponding author's responsibility.

## FUNDING

The present study was conducted without any funding and at the authors' own payment, after approval by the Council of the Department of Animal Sciences, College of Agriculture, Urmia University.

## ANIMAL WELFARE/ETHICAL APPROVAL STATEMENT

The authors confirm that the ethical policies of the journal, as noted on the journal's author guidelines page, have been adhered to and the appropriate ethical review committee approval has been received. The Veterinary Ethics Committee of Urmia University under the number of IR‐UU‐AEC for the Care and Use of Laboratory Animals were followed.

### PEER REVIEW

The peer review history for this article is available at https://publons.com/publon/10.1002/vms3.1031.

## Data Availability

The data supporting this study's findings are available from the corresponding author, Seyyed Ali Mirghelenj, upon reasonable request.
